# The effects of short-term fasting on tolerance to (neo) adjuvant chemotherapy in HER2-negative breast cancer patients: a randomized pilot study

**DOI:** 10.1186/s12885-015-1663-5

**Published:** 2015-10-05

**Authors:** Stefanie de Groot, Maaike PG Vreeswijk, Marij JP Welters, Gido Gravesteijn, Jan JWA Boei, Anouk Jochems, Daniel Houtsma, Hein Putter, Jacobus JM van der Hoeven, Johan WR Nortier, Hanno Pijl, Judith R Kroep

**Affiliations:** 1 Department of Medical Oncology, Leiden University Medical Center, Albinusdreef 2, P.O. Box 9600, 2300 RC Leiden, The Netherlands; 2 Department of Human Genetics, Leiden University Medical Center, Leiden, The Netherlands; 3 Department of Internal Medicine, Haga Hospital, The Hague, The Netherlands; 4 Department of Medical Statistics, Leiden University Medical Center, Leiden, The Netherlands; 5 Department of Endocrinology, Leiden University Medical Center, Leiden, The Netherlands

**Keywords:** Early stage breast cancer, Chemotherapy, Short-term fasting, Toxicity, DNA damage

## Abstract

**Background:**

Preclinical evidence shows that short-term fasting (STF) protects healthy cells against side effects of chemotherapy and makes cancer cells more vulnerable to it. This pilot study examines the feasibility of STF and its effects on tolerance of chemotherapy in a homogeneous patient group with early breast cancer (BC).

**Methods:**

Eligible patients had HER2-negative, stage II/III BC. Women receiving (neo)-adjuvant TAC (docetaxel/doxorubicin/cyclophosphamide) were randomized to fast 24 h before and after commencing chemotherapy, or to eat according to the guidelines for healthy nutrition. Toxicity in the two groups was compared. Chemotherapy-induced DNA damage in peripheral blood mononuclear cells (PBMCs) was quantified by the level of γ-H2AX analyzed by flow cytometry.

**Results:**

Thirteen patients were included of whom seven were randomized to the STF arm. STF was well tolerated. Mean erythrocyte- and thrombocyte counts 7 days post-chemotherapy were significantly higher (*P = 0.007,* 95 % CI 0.106-0.638 and *P = 0.00007,* 95 % CI 38.7-104, respectively) in the STF group compared to the non-STF group. Non-hematological toxicity did not differ between the groups. Levels of γ-H2AX were significantly increased 30 min post-chemotherapy in CD45 + CD3- cells in non-STF, but not in STF patients.

**Conclusions:**

STF during chemotherapy was well tolerated and reduced hematological toxicity of TAC in HER2-negative BC patients. Moreover, STF may reduce a transient increase in, and/or induce a faster recovery of DNA damage in PBMCs after chemotherapy. Larger studies, investigating a longer fasting period, are required to generate more insight into the possible benefits of STF during chemotherapy.

**Trial registration:**

ClinicalTrials.gov: NCT01304251, March 2011

**Electronic supplementary material:**

The online version of this article (doi:10.1186/s12885-015-1663-5) contains supplementary material, which is available to authorized users.

## Background

Chronic reduction of calorie intake without malnutrition reduces spontaneous cancer incidence and delays progression in a variety of tumors in rodents [1–4]. In long-term calorie restricted non-human primates, cancer incidence and mortality are reduced [5], and studies of long-term calorie restricted human subjects have shown a reduction of metabolic and hormonal factors associated with cancer risk [6–8]. Chronic calorie restriction is not practical for clinical use since it causes unacceptable weight loss in cancer patients [9]. However, brief periods of fasting may be feasible in patients and, in mice have been shown to slow cancer growth at least as effectively as chronic calorie restriction without compromising bodyweight [10–12]. Even more importantly, the effects of short-term fasting (STF) on susceptibility to chemotherapy differ between healthy somatic and cancer cells, a phenomenon called differential stress resistance (DSR) [10, 11, 13, 14]. In healthy cells, nutrient deprivation shuts down pathways promoting growth to invest energy in maintenance and repair pathways that contribute to resistance to chemotherapy [15, 16]. In contrast, tumor cells are unable to activate this protective response due to uncontrolled activation of growth pathways by oncogenic mutations. Indeed, the persistently increased growth rate of tumor cells requires abundant nutrients, and therefore, STF renders tumor cells more sensitive to chemotherapy [10–12]. Hence, STF is a promising strategy to enhance the efficacy and tolerability of chemotherapy.

In human subjects, STF is safe and well tolerated [17–19]. A case series of 10 patients with various types of cancer demonstrated that fasting in combination with chemotherapy is feasible and might reduce chemotherapy-induced side effects [20]. We conducted a randomized-controlled pilot trial to identify the effects of 48-h of STF on chemotherapy-induced side effects and hematologic parameters in breast cancer (BC) patients, who received TAC (docetaxel, doxorubicin and cyclophosphamide) chemotherapy. Furthermore, we quantified chemotherapy-induced DNA damage in peripheral blood mononucleated cells (PBMCs) by measuring γ-H2AX accumulation [21]. Upon induction of DNA double strand breaks (DSBs), H2AX is rapidly phosphorylated at the site of DNA damage [22]. γ-H2AX has been widely used to quantify DNA damage after irradiation [23–26], where the expression has been shown to be associated with healthy tissue damage [22, 27–30]. However, use of γ-H2AX as a marker for chemotherapy toxicity to healthy cells is relatively unexplored.

## Methods

### Patients

All women included in the study had a histologically confirmed diagnosis of HER2-negative stage II and III BC and were receiving (neo) adjuvant TAC-chemotherapy (see below). Eligibility criteria included age ≥ 18 years; BMI ≥19 kg/m^2^; WHO performance status 0–2; life expectancy of >3 months; adequate bone marrow function (i.e. white blood counts >3.0 × 10^9^/L, absolute neutrophil count ≥1.5 × 10^9^/l and platelet count ≥ 100 × 10^9^/l); adequate liver function (i.e. bilirubin ≤1.5 × upper limit of normal (UNL) range, ALAT and/or ASAT ≤2.5 × UNL, Alkaline Phosphatase ≤5 × UNL); adequate renal function (i.e. calculated creatinine clearance ≥50 mL/min); adequate cardiac function; absence of diabetes mellitus; absence of pregnancy or current lactation; and written informed consent. TNM classification system was used to record stage of disease in accordance with Dutch guidelines of clinical practice (http://www.oncoline.nl).

### Study design

Patients were randomized in a 1:1 ratio to fast beginning 24 h before and lasting until 24 h after start of chemotherapy (‘STF’ group) or to eat according to the guidelines for healthy nutrition with a minimum of two pieces of fruit per day (‘non-STF’ group). STF subjects were only allowed to drink water and coffee or tea without sugar. All patients kept a food diary of the consumption of food and drinks during the 24 h pre- and post-chemotherapy. All patients gave informed consent in writing. The study (NCT01304251) was conducted in accordance with the Declaration of Helsinki (October 2008) and was approved by the Ethics Committee of the LUMC in agreement with the Dutch law for medical research involving human subjects.

### Drugs

On the first day of each 3-weekly cycle (six in total), women received TAC (docetaxel 75 mg/m^2^ IV for 1 h, adriamycin 50 mg/m^2^ IV for 15 min and cyclophosphamide 500 mg/m^2^ IV for 1 h) with granulocyte-colony stimulating factor (G-CSF; pegfilgrastim 6 mg) support the day after chemotherapy administration. Patients received prophylactic dexamethasone (8 mg, BID the day before, the day of and the day after chemotherapy administration) in order to prevent fluid retention and hypersensitivity reactions. The anti-emetic agent granisetron (serotonin 5-HT_3_ receptor antagonist; 1 mg) was administered prior to chemotherapy infusion.

### Blood sampling

Venous blood samples were drawn before randomization, at a maximum of 2 weeks prior to treatment (baseline) and directly before each chemotherapy administration (pre-chemotherapy, day 0). Non-fasting blood samples were drawn from subjects in the non-STF group. The effect of fasting was determined by recording 1) metabolic parameters (insulin, glucose, insulin growth factor 1 (IGF-1), insulin growth factor binding protein 3 (IGF-BP3)); 2) endocrine parameters (thyroid-stimulating hormone (TSH), triiodothyronine (T3) and free thyroxine (FT4)); 3) hematologic parameters (erythrocyte-, thrombocytes- and leukocyte count) and 4) inflammatory response (C-Reactive Protein (CRP)). For measurement of metabolic, endocrine and inflammatory parameters , blood was collected in a serum-separating tube and for hematologic parameters, blood was collected in an EDTA tube. In addition, hematologic parameters and CRP were measured on day 7 after each chemotherapy cycle. All samples were analyzed by the accredited clinical laboratory of the LUMC.

To investigate the effect of STF on chemotherapy-induced DNA damage in PBMCs, heparinized venous blood samples (9 mL) were collected for both patient groups during each cycle just prior to chemotherapy, for some patients at 30 min after completion of chemotherapy, and on day 7 after administration. Samples were stored at room temperature until processing (in most cases directly after withdrawal or at least within 24 h).

### Toxicity

During each cycle, patients were instructed to report the experienced side effects, graded as mild, moderate or severe. Self-reported side effects, side effects documented by the physician and hematological toxicity were graded according to the Common Terminology Criteria for Adverse Events version 4.03 (CTCAE v.4.03) [31].

### Isolation of PBMCs and γ-H2AX staining

PBMCs were isolated using Ficoll Paque Plus (GE Healthcare, Uppsala, Sweden) according to the manufacturer’s instructions. Isolated PBMCs were carefully resuspended in 1 ml of Dulbecco’s Modified Eagle Medium (DMEM; Gibco) supplemented with 40 % fetal bovine serum (FBS; PAA Laboratories GmbH, Pasching, Austria) and 10 % dimethyl sulfoxide (DMSO) and divided over two cryovials. Samples were directly transferred to an isopropanol chamber and incubated at −80 °C for a minimum of 24 h to cryopreserve before they were stored in the vapor phase of liquid nitrogen.

Samples were processed batch wise, so that samples from distinct time points within each cycle were processed simultaneously for each patient. After thawing in RPMI at room temperature, PBMCs were fixed in 1.5 % formaldehyde and permealized in ice-cold methanol. Cells were washed 3 times in staining buffer (PBS with 5 % bovine serum albumin (BSA, Sigma)) and stained for 30 min on ice with anti-CD45-PerCP-Cy5.5 (1:20, BD, clone 2D1), anti-CD3-PE (1:10, BD, clone SK7), anti-CD14-AF700 (1:80, BD, clone M5E2), anti-CD15-PE CF594 (1:100, BD, clone W6D3) and anti-γ-H2AX-AF488 (1:100, Biolegend, clone 2F3), followed by another washing step. The cell acquisition was performed immediately after the staining procedure (BD LSR Fortessa Flow Cytometer analyzer, BD Bioscience, Breda, The Netherlands) and data was analyzed using BD FACS Diva Software version 6.2. Compensations were set using a mixture of anti-mouse Ig/negative control beads (BD). The CD45+ cells were gated, after which the CD3+ T lymphocytes, CD3- myeloid cells (also harboring B lymphocytes) or CD14 + CD15- monocytes were analyzed for the geomean (as measure for the intensity) of γ-H2AX.

### Statistical analysis

All parameters were tested for normality using a Kolmogorov-Smirnov test, with Bonferroni adjustment when evaluated in subgroups. Normality distributed parameters, if necessary after log transformation, were summarized as mean (and standard error (SE)) and compared using an independent samples *t*-test for independent groups or paired *t*-test for paired groups. The non-normally distributed parameters were summarized as median (and range) and compared using a Mann–Whitney test for independent groups or Wilcoxon signed rank test for paired groups. Data of different patients and different cycles were combined to test differences between time points and treatment groups. All tests were 2-tailed with a significance level of 0.05. All data were analyzed using IBM SPSS Statistics for Windows (Version 20.0. Armonk, NY: IBM Corp).

## Results

### Patient characteristics

From May 2011 until December 2012, thirteen women with early BC were included and randomized into the STF (*n* = 7) or non-STF group (*n* = 6). Patient characteristics are summarized in Table 1. In the STF arm, 42.9 % of the patients had stage III disease compared to 16.7 % of patients in the non-STF arm. Estrogen receptor status was negative for one patient in the STF group (14.3 %) and half of the patients in the non-STF group. Three patients had a Bloom-Richardson grade III tumor in the STF group and one in the non-STF group. One patient could not be graded due to the neoadjuvant chemotherapy. None of these patient characteristics was significantly different between the two groups.

**Table 1:** Patient characteristics

	STF	Non-STF	*P* Value
	(*n* = 7)	(*n* = 6)	
Median Age (range), Years	51 (47–64)	52 (44–69)	1.00
Median Body Mass Index (SEM), kg/m^2^	25.5 (3.3)	23.8 (2.4)	0.53
WHO-status			
Grade 0	6 (85.7 %)	6 (100 %)	0.34
Grade 1	1 (14.3 %)	0 (0.0 %)	
Treatment			
Adjuvant	5 (71.4 %)	3 (50.0 %)	0.43
Neo-adjuvant	2 (28.6 %)	3 (50.0 %)	
T-classification			
T1	3 (42.9 %)	2 (33.3 %)	0.94
T2	3 (42.9 %)	3 (50.0 %)	
T3	1 (14.3 %)	1 (16.7 %)	
N-classification			
N0	2 (28.6 %)	2 (33.3 %)	0.85
N+	5 (71.4 %)	4 (66.7 %)	
Stage			
II	4 (57.2 %)	5 (83.3 %)	0.31
III	3 (42.9 %)	1 (16.7 %)	
ER-status			
ER-	1 (14.3 %)	3 (50.0 %)	0.16
ER+	6 (85.7 %)	3 (50.0 %)	
PR-status			
PR-	3 (42.9 %)	4 (66.7 %)	0.39
PR+	4 (57.1 %)	2 (33.3 %)	
Grade (BR)			
1	1 (14.3 %)	1 (16.7 %)	0.44
2	2 (28.6 %)	4 (66.7 %)	
3	3 (42.9 %)	1 (16.7 %)	
Unknown	1 (14.3 %)	0 (0.0 %)	
Chemotherapy related adjustment			
No	3 (42.9 %)	3 (50.0 %)	0.80
Yes	4 (57.1 %)	3 (50.0 %)	

*STF* short-term fasting, *SEM* standard error of the mean, *ER* estrogen receptor; *PR* progesterone receptor, *BR* Bloom-Richardson

Patients were motivated to fast and the STF was well tolerated. Two patients in the STF arm withdrew from fasting after the third chemotherapy cycle: one due to pyrosis and one due to recurrent febrile neutropenia. In both patients, the side effects persisted on a normal diet during cycles 4–6. All patients finished 6 cycles of TAC. There were no significant differences in chemotherapy-related adjustments between the two groups.

### Toxicity

The most frequently observed side effects, were grade I/II and the percentage of occurrence of each side effect is recorded in Table 2. No significant differences were observed between the two patient groups. The total incidence of grade III/IV side effects that occurred in both groups is given in Table 2. The observed grade III/IV side effects were neutropenic fever, fatigue and infection (pneumonia and neutropenic enterocolitis (typhlitis)). There was no significant difference in incidence of grade III/IV side effects between the STF and non-STF group. No grade V toxicity occurred during the chemotherapy in either group.

**Table 2:** Grade I/II and grade III/IV toxicity during 6 cycles of TAC in both groups

	Grade I/II	
	STF	Non-STF
Fatigue	5 (71 %)	6 (100 %)
Infection	3 (43 %)	1 (17 %)
Mucositis	4 (57 %)	4 (67 %)
Neuropathy	5 (71 %)	3 (50 %)
Diarrhea	5 (71 %)	2 (33 %)
Dizziness	3 (43 %)	3 (50 %)
Nausea	7 (100 %)	4 (67 %)
Eye complaints	4 (57 %)	2 (33 %)
Constipation	4 (57 %)	2 (33 %)
	Grade III/IV	
Total	6	3
Neutropenic fever	2 (29 %)	2 (33 %)
Fatigue	2 (29 %)	0 (0 %)
Infection	2 (29 %)	1 (17 %)

All side effects were scored according CTCAE4.03. Each side effect was scored maximal once per patient during the course (the highest grade of occurrence was scored)

*STF* short-term fasting

### Metabolic, endocrine and inflammatory parameters

Metabolic and endocrine parameters at randomization (maximum 2 weeks before first chemotherapy cycle) and the mean or median (depending on distribution) of the day 0 values (immediately before chemotherapy infusion, when patients in the STF group had fasted for 24 h) were compared (Table 3). As no baseline values were available for three patients, no paired *t*-test could be performed, hence the deviating N values. In the STF and non-STF groups, median blood glucose values were significantly increased between the two time points (*P = 0.042* and *P = 0.043,* respectively). There was no significant difference in median insulin level between the two time points in the STF group, but in the non-STF group, the insulin level was significantly increased (*P = 0.043).* Mean IGF-1 levels were significantly decreased (*P = 0.012*) in the STF group; no change was observed in the non-STF group. IGF-BP3 levels did not change in either group. TSH was significantly reduced (*P = 0.034*) in the non-STF group, but not in the STF group. The FT4 did not change significantly over time in patients in either group.

**Table 3:** Metabolic and endocrine parameters at baseline (before randomization) and day 0 (immediately before chemotherapy infusion during the use of prophylactic dexamethasone)

Parameter	*N*	Baseline Median (range)	Day 0 (with DEX) Median (range)	In/decrease	*P* value
Glucose (3.1-6.4 mmol/L)	STF (*n* = 5)	5.2 (4.3-5.5)	6.8 (5.6-9.0)	↑	***0.042***
	Non-STF (*n* = 5)	4.8 (4.7-6.7)	7.0 (6.1-8.8)	↑	***0.043***
Insulin (0-20 mU/L)	STF (*n* = 5)	14.0 (2.0-40.0)	13.0 (6.0-36.0)	=	*0.500*
	Non-STF (*n* = 5)	2.0 (2.0-9.0)	16.0 (9.0-63.0)	↑	***0.043***
Parameter	N	Baseline Mean (SE)	Day 0 (with DEX) Mean (SE)	In/decrease	*P* value
IGF-1 (5.4-24.3 nmol/L)	STF (*n* = 4)	23.7 (2.9)	19.6 (3.3)	↓	***0.012***
	Non-STF (*n* = 5)	17.5 (3.5)	16.8 (2.8)	=	*0.634*
IGF-BP3 (2.2-5.8 mg/L)	STF (*n* = 4)	5.0 (0.5)	4.2 (0.3)	=	*0.212*
	Non-STF (*n* = 5)	4.5 (0.2)	3.9 (0.3)	=	*0.122*
TSH (0.3-4.8 mU/L)	STF (*n* = 3)	1.38 (0.26)	0.61 (0.08)	=	*0,065*
	Non-STF (*n* = 5)	1.49 (0.14)	0.42 (0.06)	↓	***0.034***
FT4 (12-22pmol/L)	STF (*n* = 3)	15.4 (0.92)	13.9 (0.94)	=	*0.117*
	Non-STF (*n* = 5)	15.0 (0.54)	14.0 (0.34)	=	*0.149*

Bold value indicates that *p* < 0.05

*DEX* dexamethasone, *IGF-1* Insulin-like growth factor 1, *IGF-BP3* insulin- like growth factor binding protein 3, *TSH* thyroid-stimulating hormone; *FT4* free thyroxine, *STF* short-term fasting, *SE* standard error

**Fig. 1:**
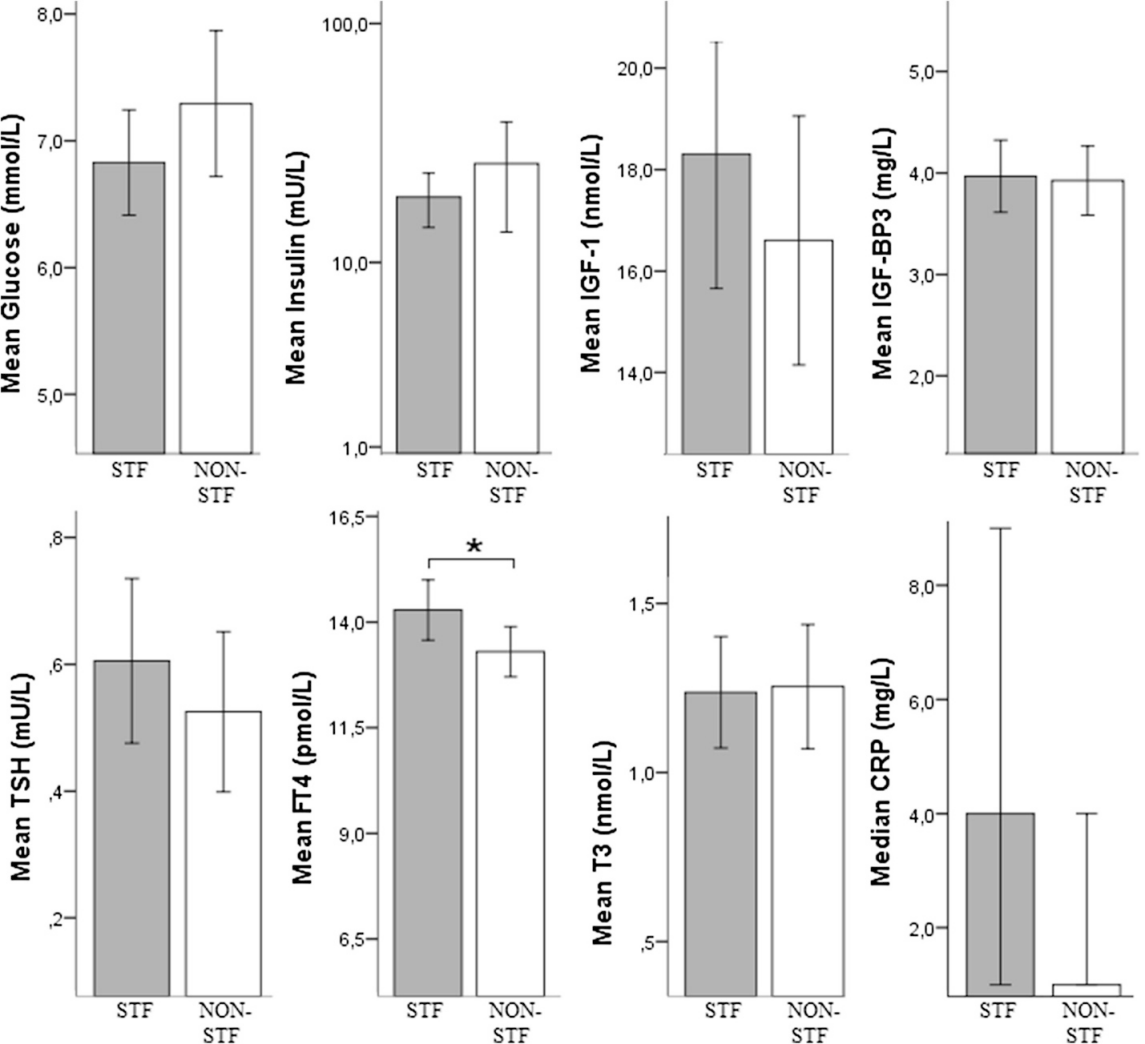
Metabolic, endocrine and inflammatory parameters on day 0 compared between STF and non-STF subjects. Values are measured on day 0 immediately before chemotherapy infusion (during the use of prophylactic dexamethasone). Mean values of different patients of different cycles (1–6) are combined to test differences between both treatment groups. * *P* value <0.05. Reference values: glucose 3.1-6.4 mmol/L; insulin 0-20 mU/L; IGF-1 5.4-24.3 nmol/L; IGF-BP3 2.2-5.8 mg/L; TSH 0.3-4.8 mU/L; FT412-22pmol/L, T31.1-3.1 nmol/L; CRP 0.0-5.0 mg/L;. IGF-1; Abbreviations: STF: short-term fasting, IGF-1:Insulin-like growth factor 1, IGF-BP3: insulin- like growth factor binding protein 3, TSH: thyroid-stimulating hormone; FT4:,free thyroxine; T3: CRP; C-reactive protein

Figure 1 shows the mean, log transformation of the mean or the median (dependent of the distribution) of day 0 metabolic, endocrine and inflammatory parameters of all cycles compared between STF and non-STF subjects. The FT4 levels were significantly higher (*P = 0.034,* 95 % CI 0.08–1.91) in the STF group compared to the non-STF group. Glucose and insulin levels appeared to be lower in the STF group compared to the non-STF group, but the difference was not statistically significant. IGF-1, IGF-BP3, TSH and T3 showed similar levels in STF and non-STF patients.

### Hematologic parameters

**Fig. 2:**
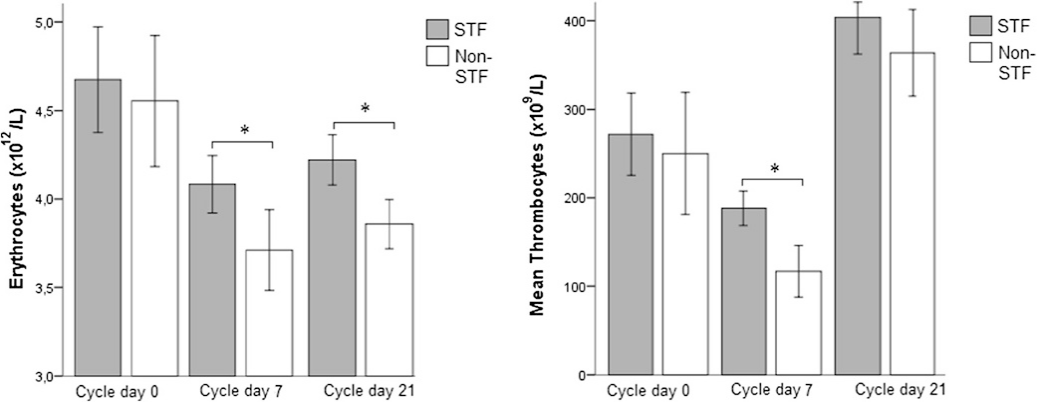
Hematologic parameters compared between both groups. Values are measured on day 0 of cycle 1 immediately before the chemotherapy infusion, on day 7 of cycle 1–5 combined and day 21 of cycle 1–5 combined. * *P* value <0.05. STF; short-term fasting, Reference values: erythrocytes 4-5*10^12^/L; thrombocytes 150-400*10^9^/L

Hematologic parameters measured on day 0 (i.e., immediately before chemotherapy infusion, when the STF group had fasted for 24 h), were similar in the two groups. Erythrocyte counts were significantly higher in the STF group during chemotherapy treatment at day 7 (*P = 0.007,* 95 % CI 0.106–0.638) and at day 21 (*P = 0.002,* 95 % CI 0.121–0.506) compared to the control group (Fig. 2). Thrombocyte counts were only significantly higher at day 7 (*P = 0.00007*, 95 % CI 38.7–104) in the STF arm compared to the non-STF arm. For leukocytes and neutrophils, no significant difference in counts was observed, both at day 7 and day 21 between STF and non-STF patients (not shown).

### DNA damage in PBMCs

No cumulative effect on DNA damage of chemotherapy was seen during the 6 cycles of TAC in CD45 + CD3+ lymphocytes, CD45 + CD14 + CD15- monocytes and CD45 + CD3- myeloid cells as no significant differences in γ-H2AX intensity were seen throughout 6 cycles, (see Additional file 1). Therefore, the measured γ-H2AX intensity from each cycle at the same time point (before chemotherapy, after 30 min, and after 7 days) was combined for analysis. The level of γ-H2AX intensity (given as geomean) measured by flow cytometry in CD45 + CD3+ lymphocytes, CD45 + CD14 + CD15- monocytes and CD45 + CD3- myeloid cells are given in Table 4. γ-H2AX intensity was increased after chemotherapy infusion in the CD45 + CD3+ lymphocytes 30 min after chemotherapy infusion in both groups and in the non-STF group after 7 days as well. In the CD45 + CD14 + CD15- monocytes no difference in γ-H2AX intensity was seen after 30 min, but after 7 days, a significant increase was seen in both groups. In the CD45 + CD3- myeloid cells, a significantly increase was seen in γ-H2AX intensity at 30 min post-chemotherapy only in the non-STF group. γ-H2AX intensity was consistently higher in CD45 + CD14 + CD15- monocytes than in CD45 + CD3+ lymphocytes and CD45 + CD3- myeloid cells.

**Table 4:** γ-H2AX intensity in CD45 + CD3+ lymphocytes, CD45 + CD14 + CD15- monocytes and CD45 + CD3-myeloid cells

Parameter	N	Before CT Day 0 Mean (SE)	30 minutes after CT Day 0 Mean (SE)	Increase	*P* value
CD45 + CD3+ lymphocytes	STF (*n* = 14)	75.5 (4.7)	89.5 (6.5)	↑	***0.020***
	Non-STF (*n* = 6)	78.8 (5.6)	95.7 (5.9)	↑	***0.001***
CD45 + CD14 + CD15- monocytes	STF (*n* = 12)	162.2 (11.9)	192.5 (14.3)	=	*0.055*
	Non-STF (*n* = 6)	180.8 (15.6)	206.2 (20.8)	=	*0.051*
CD45 + CD13- myeloid cells	STF (*n* =14)	104.0 (7.0)	109.5 (8.4)	=	*0.594*
	Non-STF (*n* = 6)	109.0 (7.8)	123.0 (6.7)	↑	***0.009***
Parameter	N	Before CT Day 0 Median (range)	7 days after CT Median (range)	Increase	*P* value
CD45 + CD3+ lymphocytes	STF (*n* =16)	75.5 (49–157)	83.0 (64–141)	=	*0.109*
	Non-STF (*n* = 9)	78.0 (47–102)	90.0 (71–114)	↑	***0.015***
CD45 + CD14 + CD15- monocytes	STF (*n* = 14)	157.0 (114–231)	186.5 (132–295)	↑	***0.035***
	Non-STF (*n* = 8)	203.5 (116–273)	258.5 (183–319)	↑	***0.021***
CD45 + CD13- myeloid cells	STF (*n* = 16)	106.0 (71–173)	84.0 (65–145)	=	*0.379*
	Non-STF (*n* = 9)	88.0 (49–137)	88.0 (74–119)	=	*0.477*

Paired comparison between pre- and post- chemotherapy (30 minutes and 7 days; median of 6 cycles of TAC) for the different cell types. γ-H2AX intensity is given as mean and median depending on the distribution

Bold value indicates that *p* < 0.05. 95 % CI; 95 % confidence interval. *P* values are given for differences of intensity of γ-H2AX between pre- and post-chemotherapy

## Discussion

This is the first randomized pilot study to explore the effects of 48 h STF on the side effects of chemotherapy in early BC patients. Only one study to date [20] has examined the effects of fasting on chemotherapy-induced side effects in cancer patients, but therein the patients served as their own controls and had various tumor types and treatment protocols. The main findings of our study were that STF was well-tolerated, safe and had beneficial effects on hematologic toxicity and possibly on DNA damage in healthy cells (lymphocytes and myeloid cells). 

Although STF was well tolerated, two patients withdrew from STF after 3 cycles of chemotherapy after experiencing a side effect (pyrosis and recurrent febrile neutropenia, respectively). Since these side effects persisted in both patients during the subsequent 3 cycles of chemotherapy without STF, they may not be related to STF. All patients finished their treatment schedule of 6 cycles of TAC and no significant difference in occurrence of chemotherapy-related adjustments were found between the two groups. The side effect profile of the TAC protocol seen in this study was consistent with the existing literature [32–34]. STF had no beneficial effect on patient-reported side effects in this study. This may be explained by the large variability of side effects between patients, which may be attributable to occurrence of symptom clusters and pharmacogenomics [35, 36]. This may have masked any beneficial effects of STF. Additionally, the relatively short period of fasting (48 h) may explain the lack of benefit in terms of side effects: previous studies have shown that a longer fasting period is required to cause major changes in IGF-1 levels [20, 37]. Reduction of plasma IGF-1 levels is a critical mediator of differential stress resistance in response to nutrient restriction (see below).

γ-H2AX phosphorylation indicates the presence of double-strand DNA breaks and could serve as a marker for chemotherapy toxicity in healthy cells, as seen in a phase I/II trial with patients treated with chemotherapy and belinostat [38]. We measured the induction of chemotherapy-induced DNA damage in PBMCs by phosphorylation of H2AX (i.e. γ-H2AX). The level of γ-H2AX in CD45 + CD3+ lymphocytes was increased after 30 min in both groups. After 7 days, γ-H2AX accumulation remained increased in the non-STF group only, suggesting that STF promotes the recovery of chemotherapy-induced DNA damage in these cells. In CD45 + CD3- myeloid cells, the level of γ-H2AX was increased after 30 min in the non-STF group, but not in the STF group, suggesting STF protected these cells against the induction of DNA damage by chemotherapy. As these myeloid cells may harbor the antigen-presenting cells required for induction of an effective anti-tumor immune response, this result warrants further study [39]. Moreover, the relation of this finding with the clinical benefit of STF still needs to be established.

The significantly higher erythrocyte and thrombocyte counts observed after chemotherapy in the STF group could be explained by decreased breakdown of circulating cells and/or less severe bone marrow suppression. This supports the hypothesis that STF may protect against chemotherapy-associated hematological toxicity. No significant difference in leukocyte and neutrophil counts was seen. This could be explained by the use of pegfilgrastim, which acts to increase the production of white blood cells in bone marrow and may therefore prevent a decrease in leukocyte counts in response to chemotherapy.

Plasma glucose levels increased and insulin levels remained constant in response to STF. The use of dexamethasone may explain this phenomenon [40–42]. Dexamethasone was administered for anti-emesis, reduction of fluid retention and dampening of hypersensitivity reactions in response to docetaxel [43]. However, the metabolic effects of dexamethasone may have attenuated the benefits of STF. In the absence of dexamethasone, STF reduces circulating glucose, insulin and IGF-1 levels [19, 44]. A decrease in IGF-1 affects other factors (e.g. Akt, Ras and mammalian target of rapamycin (mTOR)) to down-regulate cell growth and proliferation [45–47]. Reduction of IGF-1 is one of the key mediators of the protective effects of STF in healthy cells [44]. Although fasting modestly reduced plasma IGF-1 concentrations in the current trial, the concomitant use of dexamethasone probably attenuated the decline and thereby probably counteracted the beneficial impact of the dietary intervention.

Our study has some limitations. The most obvious limitation of our study is the small sample size, which may have limited the power of the study and precludes firm statistical conclusions. Moreover, as high dose dexamethasone induces insulin resistance, compensatory hyperinsulinemia and hyperglycemia, its prophylactic use may have counteracted the beneficial effects of STF. Therefore the use of this drug warrants further study for future clinical trials with STF. Finally, as DNA damage is repaired rapidly [48], our protocol may not be rapid enough to obtain a reliable quantification. Therefore, a consistent and rapid protocol for the isolation and fixation of PBMCs immediately after blood withdrawal should be applied in future studies to allow for reliable quantification of damage induced by chemotherapy.

Larger randomized trials such as the DIRECT study (NCT02126449) are now ongoing to evaluate the impact of STF on tolerance to and efficacy of neoadjuvant chemotherapy in women with stage II or III BC. Because it is likely that the positive effects of STF will be enhanced if the period of fasting is prolonged [37, 49], a very low calorie, low protein fasting mimicking diet (FMD) is used to ease the burden of prolonged fasting [50]. Prophylactic dexamethasone will be omitted in the FMD arm during the first 4 chemotherapy cycles to reduce its potentially counteractive metabolic effects. Moreover, blood will be processed immediately after sampling to prevent potential recovery of DNA damage.

## Conclusions

We demonstrate for the first time that STF is feasible for a period of 48 h during chemotherapy in a homogeneous group of patients with early breast cancer. This study provides evidence that STF attenuates bone marrow toxicity in these patients and reduces chemotherapy-induced DNA damage in PBMCs and/or accelerate its recovery. A larger trial with a longer fasting period is ongoing to investigate the possible benefits of STF during chemotherapy.

## Additional file

Additional file 1: Table S1. Median of γ-H2AX geomean intensity in CD45 + CD3+ lymphocytes, CD45 + CD14 + CD15- monocytes and CD45 + CD3- myeloid cells among the six cycles tested with the median test, testing for differences of γ-H2AX between cycles. (DOCX 14 kb)

## Abbreviations

BC: Breast cancer
CRP: C-Reactive protein
DSBs: Double-strand breaks
DSR: Differential stress resistance
FT4: Free thyroxine
G-CSF: Granulocyte-colony stimulating factor
IGF-1: Insulin growth factor 1
IGF-BP3: Insulin growth factor binding protein 3
PBMCs: Peripheral blood mononuclear cells
STF: Short-term fasting
T3: Triiodothyronine
TAC: Docetaxel, doxorubicin and cyclophosphamide
TSH: Thyroid-stimulating hormone
UNL: Upper limit of normal

## References

[1] Sheldon WG, Bucci TJ, Hart RW, Turturro A (1995). Age-related neoplasia in a lifetime study of ad libitum-fed and food-restricted B6C3F1 mice. Toxicol Pathol.

[2] Mulrooney TJ, Marsh J, Urits I, Seyfried TN, Mukherjee P (2011). Influence of caloric restriction on constitutive expression of NF-kappaB in an experimental mouse astrocytoma. PLoS One.

[3] De Lorenzo MS, Baljinnyam E, Vatner DE, Abarzua P, Vatner SF, Rabson AB (2011). Caloric restriction reduces growth of mammary tumors and metastases. Carcinogenesis.

[4] Weindruch R, Walford RL (1982). Dietary restriction in mice beginning at 1 year of age: effect on life-span and spontaneous cancer incidence. Science.

[5] Colman RJ, Anderson RM, Johnson SC, Kastman EK, Kosmatka KJ, Beasley TM (2009). Caloric restriction delays disease onset and mortality in rhesus monkeys. Science.

[6] Fontana L, Weiss EP, Villareal DT, Klein S, Holloszy JO (2008). Long-term effects of calorie or protein restriction on serum IGF-1 and IGFBP-3 concentration in humans. Aging Cell.

[7] Renehan AG, Zwahlen M, Minder C, O’Dwyer ST, Shalet SM, Egger M (2004). Insulin-like growth factor (IGF)-I, IGF binding protein-3, and cancer risk: systematic review and meta-regression analysis. Lancet.

[8] Walford RL, Mock D, Verdery R, MacCallum T (2002). Calorie restriction in biosphere 2: alterations in physiologic, hematologic, hormonal, and biochemical parameters in humans restricted for a 2-year period. J Gerontol A Biol Sci Med Sci.

[9] Doyle C, Kushi LH, Byers T, Courneya KS, Demark-Wahnefried W, Grant B (2006). Nutrition and physical activity during and after cancer treatment: an American Cancer Society guide for informed choices. CA Cancer J Clin.

[10] Lee C, Raffaghello L, Brandhorst S, Safdie FM, Bianchi G, Martin-Montalvo A (2012). Fasting cycles retard growth of tumors and sensitize a range of cancer cell types to chemotherapy. Sci Transl Med.

[11] Raffaghello L, Lee C, Safdie FM, Wei M, Madia F, Bianchi G (2008). Starvation-dependent differential stress resistance protects normal but not cancer cells against high-dose chemotherapy. Proc Natl Acad Sci U S A.

[12] Safdie F, Brandhorst S, Wei M, Wang W, Lee C, Hwang S (2012). Fasting enhances the response of glioma to chemo- and radiotherapy. PLoS One.

[13] Laviano A, Rossi FF (2012). Toxicity in chemotherapy--when less is more. N Engl J Med.

[14] Longo VD, Mattson MP (2014). Fasting: molecular mechanisms and clinical applications. Cell Metab.

[15] Fontana L, Partridge L, Longo VD (2010). Extending healthy life span--from yeast to humans. Science.

[16] Bishop NA, Guarente L (2007). Genetic links between diet and lifespan: shared mechanisms from yeast to humans. Nat Rev Genet.

[17] Maccario M, Aimaretti G, Grottoli S, Gauna C, Tassone F, Corneli G (2001). Effects of 36 hour fasting on GH/IGF-I axis and metabolic parameters in patients with simple obesity. Comparison with normal subjects and hypopituitary patients with severe GH deficiency. Int J Obes Relat Metab Disord.

[18] Wijngaarden MA, van der Zon GC, Willems van Dijk KW, Pijl H, Guigas B (2013). Effects of prolonged fasting on AMPK signaling, gene expression and mitochondrial respiratory-chain content in skeletal muscle from lean and obese individuals. Am J Physiol Endocrinol Metab.

[19] Bergman BC, Cornier MA, Horton TJ, Bessesen DH (2007). Effects of fasting on insulin action and glucose kinetics in lean and obese men and women. Am J Physiol Endocrinol Metab.

[20] Safdie FM, Dorff T, Quinn D, Fontana L, Wei M, Lee C (2009). Fasting and cancer treatment in humans: a case series report. Aging (Albany NY).

[21] Kuo LJ, Yang LX (2008). Gamma-H. In Vivo.

[22] Rube CE, Grudzenski S, Kuhne M, Dong X, Rief N, Lobrich M (2008). DNA double-strand break repair of blood lymphocytes and normal tissues analysed in a preclinical mouse model: implications for radiosensitivity testing. Clin Cancer Res.

[23] Taneja N, Davis M, Choy JS, Beckett MA, Singh R, Kron SJ (2004). Histone H2AX phosphorylation as a predictor of radiosensitivity and target for radiotherapy. J Biol Chem.

[24] Klokov D, MacPhail SM, Banath JP, Byrne JP, Olive PL (2006). Phosphorylated histone H2AX in relation to cell survival in tumor cells and xenografts exposed to single and fractionated doses of X-rays. Radiother Oncol.

[25] Redon CE, Dickey JS, Bonner WM, Sedelnikova OA (2009). gamma-H2AX as a biomarker of DNA damage induced by ionizing radiation in human peripheral blood lymphocytes and artificial skin. Adv Space Res.

[26] Olive PL, Banath JP (2004). Phosphorylation of histone H2AX as a measure of radiosensitivity. Int J Radiat Oncol Biol Phys.

[27] Li P, Du CR, Xu WC, Shi ZL, Zhang Q, Li ZB (2013). Correlation of dynamic changes in gamma-H2AX expression in peripheral blood lymphocytes from head and neck cancer patients with radiation-induced oral mucositis. Radiat Oncol.

[28] Fleckenstein J, Kuhne M, Seegmuller K, Derschang S, Melchior P, Graber S (2011). The impact of individual in vivo repair of DNA double-strand breaks on oral mucositis in adjuvant radiotherapy of head-and-neck cancer. Int J Radiat Oncol Biol Phys.

[29] Rube CE, Fricke A, Schneider R, Simon K, Kuhne M, Fleckenstein J (2010). DNA repair alterations in children with pediatric malignancies: novel opportunities to identify patients at risk for high-grade toxicities. Int J Radiat Oncol Biol Phys.

[30] Bourton EC, Plowman PN, Smith D, Arlett CF, Parris CN (2011). Prolonged expression of the gamma-H2AX DNA repair biomarker correlates with excess acute and chronic toxicity from radiotherapy treatment. Int J Cancer.

[31] http://evs.nci.nih.gov/ftp1/CTCAE/CTCAE_4.03_2010-06-14_QuickReference_5x7.pdf: Internet 2015.

[32] Hatam N, Ahmadloo N, Ahmad Kia DA, Bastani P, Askarian M (2011). Quality of life and toxicity in breast cancer patients using adjuvant TAC (docetaxel, doxorubicin, cyclophosphamide), in comparison with FAC (doxorubicin, cyclophosphamide, 5-fluorouracil). Arch Gynecol Obstet.

[33] von Minckwitz G, Kummel S, du Bois A, Eiermann W, Eidtmann H, Gerber B (2008). Pegfilgrastim +/− ciprofloxacin for primary prophylaxis with TAC (docetaxel/doxorubicin/cyclophosphamide) chemotherapy for breast cancer. Results from the GEPARTRIO study. Ann Oncol.

[34] Charehbili A, van de Ven S, Smit VT, Kranenbarg EM, Hamdy NA, Putter H (2014). Addition of zoledronic acid to neoadjuvant chemotherapy does not enhance tumor response in patients with HER2 negative stage II/III breast cancer: the NEOZOTAC trial (BOOG 2010–01). Ann Oncol.

[35] Evans WE, McLeod HL (2003). Pharmacogenomics--drug disposition, drug targets, and side effects. N Engl J Med.

[36] Kim HJ, McGuire DB, Tulman L, Barsevick AM (2005). Symptom clusters: concept analysis and clinical implications for cancer nursing. Cancer Nurs.

[37] Thissen JP, Ketelslegers JM, Underwood LE (1994). Nutritional regulation of the insulin-like growth factors. Endocr Rev.

[38] Thomas A, Rajan A, Szabo E, Tomita Y, Carter CA, Scepura B (2014). A Phase I/II trial of belinostat in combination with cisplatin, doxorubicin and cyclophosphamide in thymic epithelial tumors: a clinical and translational study. Clin Cancer Res.

[39] Tesniere A, Apetoh L, Ghiringhelli F, Joza N, Panaretakis T, Kepp O (2008). Immunogenic cancer cell death: a key-lock paradigm. Curr Opin Immunol.

[40] Grill V, Pigon J, Hartling SG, Binder C, Efendic S (1990). Effects of dexamethasone on glucose-induced insulin and proinsulin release in low and high insulin responders. Metabolism.

[41] Hickish T, Astras G, Thomas P, Penfold S, Purandare L, Hickish TF (2009). Glucose intolerance during adjuvant chemotherapy for breast cancer. J Natl Cancer Inst.

[42] Matsumoto K, Yamasaki H, Akazawa S, Sakamaki H, Ishibashi M, Abiru N (1996). High-dose but not low-dose dexamethasone impairs glucose tolerance by inducing compensatory failure of pancreatic beta-cells in normal men. J Clin Endocrinol Metab.

[43] Sanofi-Aventis. Taxotere (docetaxel) package insert. Bridgewater, NJ: 2007.

[44] Lee C, Longo VD (2011). Fasting vs dietary restriction in cellular protection and cancer treatment: from model organisms to patients. Oncogene.

[45] Hardie DG, Ross FA, Hawley SA (2012). AMPK: a nutrient and energy sensor that maintains energy homeostasis. Nat Rev Mol Cell Biol.

[46] Laplante M, Sabatini DM (2012). mTOR signaling in growth control and disease. Cell.

[47] Zoncu R, Efeyan A, Sabatini DM (2011). mTOR: from growth signal integration to cancer, diabetes and ageing. Nat Rev Mol Cell Biol.

[48] Scarpato R, Castagna S, Aliotta R, Azzara A, Ghetti F, Filomeni E (2013). Kinetics of nuclear phosphorylation (gamma-H2AX) in human lymphocytes treated in vitro with UVB, bleomycin and mitomycin C. Mutagenesis.

[49] Snel M, Wijngaarden MA, Bizino MB, van der Grond J, Teeuwisse WM, van Buchem MA (2012). Food cues do not modulate the neuroendocrine response to a prolonged fast in healthy men. Neuroendocrinology.

[50] Brandhorst S, Choi IY, Wei M, Cheng CW, Sedrakyan S, Navarrete G (2015). A periodic diet that mimics fasting promotes multi-system regeneration, enhanced cognitive performance, and health span. Cell Metab.

