# Supplementary material for: The effects of short-term fasting on tolerance to (neo) adjuvant chemotherapy in HER2-negative breast cancer patients: a randomized pilot study
Source: BMC Cancer. 2015 Oct 5;15:652. doi: 10.1186/s12885-015-1663-5 (PMC4595051; doi:10.1186/s12885-015-1663-5)
Supplement: Additional file 1: Table S1. — Median of γ-H2AX geomean intensity in CD45 + CD3+ lymphocytes, CD45 + CD14 + CD15- monocytes and CD45 + CD3- myeloid cells among the six cycles tested with the median test, testing for differences of γ-H2AX between cycles. (DOCX 14 kb) [file 12885_2015_1663_MOESM1_ESM.docx]

*Supplementary Table 1. Median of γ-H2AX geomean intensity in CD45+CD3+ lymphocytes, CD45+CD14+CD15- monocytes and CD45+CD3- myeloid cells among the six cycles tested with the median test, testing for differences of γ-H2AX between cycles.*

|  | ***CD45+CD3+ lymphocytes*** |  | ***CD45+CD14+***  ***CD15- monocytes*** |  | ***CD45+CD3- myeloid cells*** |  |
| --- | --- | --- | --- | --- | --- | --- |
| ***Time point*** | ***Median γ-H2AX intensity*** | ***P value**** | ***Median H2AX intensity*** | ***P value**** | ***Median γ-H2AX intensity*** | ***P Value**** |
| ***before CT Day 0*** | 78.0 | 0.265 | 156.5 | 0.147 | 106.0 | 0.953 |
| ***30 minutes after CT Day 0*** | 97.0 | 0.931 | 177.5 | 0.502 | 123.0 | 0.931 |
| ***7 days after CT*** | 86.0 | 0.440 | 181.5 | 0.602 | 84.0 | 0.514 |

*P values* given for differences of intensity of γ-H2AX tested among the six cycles.*
